# Body Composition Analysis in Postoperative Breast Cancer Patients Undergoing Chemotherapy and Its Association with Physical Activity and Quality of Life: A Longitudinal Pilot Study

**DOI:** 10.3390/nu17213352

**Published:** 2025-10-24

**Authors:** Joanna Grupińska, Marika Wlazło, Mateusz Grajek, Magdalena Budzyń, Ewa Malchrowicz-Mośko, Tomasz Jurys

**Affiliations:** 1 Chair and Department of Medical Chemistry and Laboratory Medicine, Poznan University of Medical Sciences, 8 Rokietnicka, 60806 Poznan, Poland; jgrupinska@ump.edu.pl (J.G.); magdalenabudzyn@ump.edu.pl (M.B.); 2 Hospital Pharmacy, Greater Poland Cancer Centre, 15 Garbary, 61866 Poznan, Poland; 3 Department of Cardiovascular Disease Prevention, Faculty of Public Health in Bytom, Medical University of Silesia in Katowice, 41902 Bytom, Poland; d201206@365.sum.edu.pl; 4 Department of Public Health, Faculty of Public Health in Bytom, Medical University of Silesia in Katowice, Piekarska 18, 41902 Bytom, Poland; mgrajek@sum.edu.pl; 5 Faculty of Physical Education, Józef Piłsudski University of Physical Education, Marymoncka 34, 00-968 Warsaw, Poland; ewa.malchrowicz@awf.edu.pl; 6 Department of Rehabilitation, Faculty of Health Sciences in Katowice, Medical University of Silesia in Katowice, Medyków 12, 40751 Katowice, Poland

**Keywords:** breast cancer, chemotherapy, body composition, physical activity

## Abstract

**Background/Objectives**: Breast cancer survivors often experience adverse body composition changes and reduced quality of life (QoL) after chemotherapy. This study aimed to assess changes in body composition in postoperative breast cancer patients undergoing chemotherapy and to examine their associations with physical activity and QoL. **Methods**: This longitudinal observational pilot study included two repeated assessments (after surgery and before the third chemotherapy cycle – six weeks period). Sixty women (mean age 57 ± 10 years) who had undergone breast cancer surgery and were scheduled for chemotherapy were assessed twice: after surgery and prior to the third chemotherapy cycle. Body composition was analyzed using anthropometric and bioelectrical impedance methods. Physical activity was evaluated with the International Physical Activity Questionnaire–Long Form (IPAQ-L), while QoL was measured with the World Health Organization Quality of Life–bref version (WHOQOL-BREF) questionnaire as well. **Results**: During chemotherapy, participants showed significant increases in body weight (*p* = 0.001), BMI (*p* = 0.001), and muscle mass (*p* = 0.001), with stable fat percentage. Physical activity levels improved overall, particularly in moderate activity (*p* = 0.001), while sedentary time decreased (*p* = 0.020). QoL remained generally stable, with significant improvement in the environmental domain (*p* = 0.028). Higher fat percentage correlated negatively with physical (*p* = 0.040) and social (*p* = 0.049) QoL, while BMI correlated inversely with psychological well-being (*p* = 0.020). Waist-to-hip ratio was also negatively associated with psychological QoL (*p* = 0.017). **Conclusions**: Vigorous activity showed an association with more favorable body composition, whereas sedentary behavior correlated with higher BMI and muscle mass.

## 1. Introduction

Breast cancer is the most commonly diagnosed malignant neoplasm among women worldwide. According to global statistics, in 2020 approximately 2.3 million new cases of breast cancer were recorded, accounting for 11.7% of all newly diagnosed cancers. In the same year, about 685,000 women died of breast cancer globally, representing roughly 6.9% of all cancer-related deaths. Thanks to advances in prevention and therapy, survival rates have significantly improved. In developed countries, breast cancer is increasingly detected at an early stage—the 5-year relative survival rate for localized disease currently reaches approximately 99%. The growing effectiveness of treatment has resulted in a steadily increasing population of women who have completed therapy for breast cancer and live as survivors [1].

Despite improved prognosis, many breast cancer survivors struggle with the long-term consequences of the disease and its treatment. Chemotherapy can cause numerous chronic complications that diminish quality of life. Among the most common are chronic pain, reduced mobility and strength of the upper limb on the operated side, fatigue (including cancer-related fatigue syndrome), metabolic disturbances (e.g., dyslipidemia), as well as weight gain, obesity, premature menopause, and lymphedema. These consequences negatively impact patients’ quality of life after the completion of oncological treatment, despite biological cure of the disease. Therefore, in survivorship care (secondary prevention), increasing emphasis is placed on monitoring health status and implementing interventions that support full recovery of physical function [2].

Among the long-term effects of breast cancer treatment, adverse changes in body composition occupy a special place. Numerous studies indicate that women after chemotherapy tend to gain weight—with increases in fat mass accompanied by reductions in lean body mass (so-called sarcopenic obesity). It is estimated that nearly 50% of breast cancer survivors are overweight or obese, and many patients experience weight gain during hormonal therapy or immediately following chemotherapy. Excess body weight in this group has serious clinical implications [3]. Obese breast cancer survivors are more likely to report poorer health-related quality of life. Furthermore, persistent overweight or obesity after completion of oncological therapy correlates with a higher risk of cancer recurrence and increased overall mortality compared with patients of normal body weight. For this reason, standard recommendations for breast cancer survivors emphasize the importance of weight management. Weight reduction is treated as a component of secondary cancer prevention, aimed at improving both prognosis and quality of life [4].

In recent years, a growing number of studies have focused on the role of physical activity during recovery after breast cancer. Regular exercise is now considered a safe and recommended element of oncological rehabilitation. Just over a decade ago, oncologists often advised patients to adopt a more sedentary lifestyle during treatment, fearing that physical exertion could harm an already weakened body. Today, however, the opposite approach prevails—numerous studies have demonstrated that regular physical activity provides significant benefits to cancer survivors [4]. The American College of Sports Medicine (ACSM), in its official position, emphasizes that exercise is safe for cancer survivors and, in fact, recommends avoiding inactivity altogether. Survivors are encouraged to meet the same physical activity standards as the general population—namely, at least 150 min of moderate-intensity aerobic exercise per week, plus two sessions of strength training targeting the major muscle groups. Naturally, exercise programs should be adapted to the patient’s condition (taking into account prior surgeries, risk of lymphedema, etc.), but avoiding prolonged inactivity and gradual return to exercise are crucial [5].

The relationship between body composition, physical activity, and quality of life in women after breast cancer has been the subject of intensive research. The results to date clearly indicate the benefits of adopting a healthy lifestyle in this population. Maintaining a healthy body weight (or reducing excess weight) and engaging in regular physical activity in line with recommendations are key factors that support improved quality of life and overall health in women after chemotherapy. Implementing exercise programs for breast cancer survivors, alongside health education on nutrition and weight control, is now recognized as an essential component of comprehensive oncological care. Such a holistic approach not only prolongs disease-free survival but, above all, improves quality of life, enabling women to return to full family, professional, and social activity.

The aim of the study is to assess body composition in postoperative breast cancer patients undergoing chemotherapy and to examine its association with physical activity levels and quality of life. 

## 2. Materials and Methods

### 2.1. Participants

The research was carried out with female patients admitted to the Department of Surgical Oncology of Gastrointestinal Diseases at the Greater Poland Cancer Center (Wielkopolskie Centrum Onkologii) in Poznań. The participants were women with histopathologically verified breast cancer who had undergone radical surgery and were then scheduled to receive systemic chemotherapy in the adjuvant setting. Eligible participants were adult women between 40 and 80 years of age, free from cognitive impairments, and who had signed informed consent to take part in the study. Women were excluded if they had concomitant endocrine or metabolic disorders (such as thyroid disease or Cushing’s syndrome), chronic kidney or liver conditions, autoimmune illnesses, insulin-dependent diabetes, a diagnosis of another cancer within the previous five years, recurrent breast cancer, or unstable cardiovascular health.

Of the 69 initially recruited participants, 60 completed the study. The participants’ average age was 57 years (±10). According to the World Health Organization Performance Status scale, all patients scored either 0 or 1, reflecting normal activity or only slight limitations in strenuous physical tasks. The majority had tumors classified as histological grade G2 or lower (45.0%) or grade G3 (48.3%). Overexpression of the HER2 receptor was found in 20 of the cases (33.3%), whereas the remaining 40 (66.7%) were HER2-negative. In more than half of the women, the primary tumor measured ≤ 2 cm, and nearly half showed no lymph node metastasis.

### 2.2. Study Design

This was a pilot, exploratory, longitudinal observational study intended to generate preliminary data and hypotheses for larger confirmatory trials with two repeated assessments. No experimental intervention was applied, and participants continued with their standard oncological treatment plans. Each woman was evaluated at two stages: first, after surgery but before starting chemotherapy, and again roughly six weeks later, just before the third chemotherapy cycle. Data collection encompassed body measurements, evaluation of physical activity and quality of life, along with an analysis of medical records. No control group was included; therefore, observed changes cannot be attributed solely to chemotherapy effects. Possible confounding factors, such as postoperative recovery, were acknowledged and discussed as potential sources of bias.

### 2.3. Assessment

Anthropometric measurements were collected in accordance with standardized clinical procedures. Body weight was determined to the nearest 0.1 kg using a calibrated electronic scale, and height was measured to the nearest 0.5 cm with a stadiometer. Body mass index (BMI) was then calculated as body weight in kilograms divided by height in meters squared (kg/m^2^). Waist and hip circumferences were measured with a non-elastic tape, placed at the midpoint between the lowest rib and the iliac crest for the waist, and at the level of the greater trochanters for the hip. Information on tumor size, histological grade, and HER2 receptor status was obtained from medical records as reported by the treating oncologists.

The World Health Organization Quality of Life–bref version (WHOQOL-BREF) questionnaire consists of 26 items divided into four domains: physical health, psychological health, social relationships, and environment, along with two general questions assessing overall quality of life and health. Each item is scored on a five-point Likert scale (1–5), and the results are converted to a 0–100 scale [6,7]. The physical domain encompasses aspects such as mobility, daily functioning, energy levels, pain, and sleep. The psychological domain evaluates body image, emotions, self-esteem, and cognitive abilities. The social domain addresses interpersonal relationships, social support, and sexual life, while the environmental domain considers access to resources, safety, healthcare services, housing conditions, educational opportunities, leisure, and transportation [6,7].

Physical activity was evaluated using the International Physical Activity Questionnaire–Long Form (IPAQ-L). This instrument measures activity across four areas: work-related tasks, transportation (such as walking or cycling), household and yard duties, and leisure-time activities performed during the previous week. The Polish version of the questionnaire was developed in line with international standards, ensuring cross-population comparability, and maintains the psychometric quality of the original, demonstrating high test–retest reliability (r = 0.7–0.8) and acceptable criterion validity when compared with accelerometer data [8,9]. Overall physical activity was reported as MET-minutes per week, which allowed categorization into low, moderate, or high activity levels.

### 2.4. Ethics

The study protocol received approval from the Bioethics Committee of the Poznań University of Medical Sciences (Resolution No. 1016). All participants were informed about the study’s objectives and procedures and gave written informed consent, consistent with the principles of the Declaration of Helsinki.

### 2.5. Statistical Analysis

Data were analyzed using StatSoft Statistica version 13.3. Continuous variables were expressed as mean ± standard deviation (SD) or as median with interquartile range, depending on data distribution. Comparisons between the two measurement points were performed with the paired Student’s *t*-test or, in the case of nonparametric data, the Wilcoxon signed-rank test. Categorical variables were examined using the chi-square test. The Shapiro–Wilk test was applied to verify the normality of distributions.

In addition to descriptive and comparative analyses (paired *t*-test or Wilcoxon test for continuous variables, chi-square or Fisher’s exact test for categorical variables), correlation analyses were carried out to explore relationships between psychological outcomes and physical activity domains. Given the non-normal distribution of IPAQ-L results, Spearman’s rank correlation coefficients (ρ) were calculated. The threshold for statistical significance was set at *p* < 0.05.

## 3. Results

### 3.1. Physical Activity

**Figure 1:**
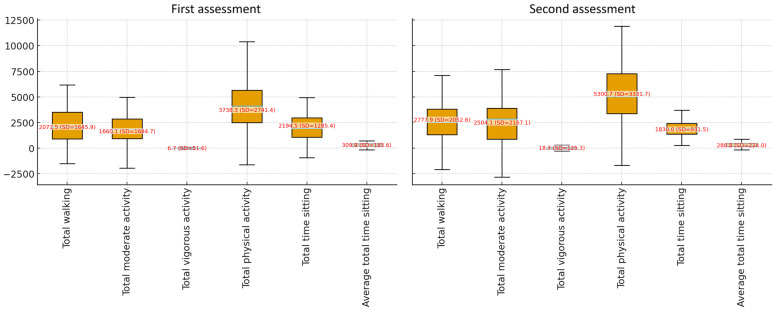
Results for IPAQ–L domains (N = 60).

In the analysis of physical activity and sitting time between the first and second measurements, clear changes were observed in both mean values and statistical significance. For walking, the mean increased from 2071.5 MET-min/week (SD = 1645.9) in the first measurement to 2777.9 MET-min/week (SD = 2052.6) in the second. The median shifted from 2079.0 to 2194.5, with maximum values reaching 11,088.0. The difference was close to statistical significance (*p* = 0.057), indicating an upward trend. Moderate activity showed a significant increase, with the mean rising from 1660.1 (SD = 1684.7) to 2504.1 (SD = 2167.1), and the median increasing from 1260.0 to 2370.0. This change was statistically significant (*p* = 0.001), confirming a substantial improvement in moderate activity levels. Vigorous activity remained low, with the mean increasing only from 6.7 (SD = 51.6) to 18.7 (SD = 125.3), while the median remained at 0.0 in both measurements. The difference was not significant (*p* = 0.179), suggesting no notable change in vigorous activity. For total physical activity, there was a marked increase from 3738.3 (SD = 2741.4) to 5300.7 (SD = 3331.7), with the median rising from 3543.0 to 5242.5. Maximum values reached as high as 16,608.0 MET-min/week in the second measurement. The difference was statistically significant (*p* = 0.002), confirming a clear improvement in overall physical activity levels. In terms of sedentary behavior, total sitting time decreased from 2194.5 min/week (SD = 1285.4) in the first measurement to 1830.0 min/week (SD = 931.5) in the second. The median dropped from 1875.0 to 1680.0, with this difference being statistically significant (*p* = 0.020), indicating a meaningful reduction in sitting time. Average daily sitting time also declined, from 309.9 (SD = 183.6) to 288.8 (SD = 224.0), with the median shifting from 266.0 to 240.0. This change was borderline significant (*p* = 0.062), pointing to a downward trend. Figure 1 shows IPAQ–L results.

### 3.2. Quality of Life

Analysis of WHOQOL-BREF results showed that women’s quality of life remained generally stable across most domains during the early stages of chemotherapy (Table 1). Physical health scores were moderate and demonstrated minimal change between the two assessments (−0.5, *p* > 0.05), indicating ongoing difficulties with mobility, energy, and daily activities. Psychological well-being was comparatively higher, with a small, non-significant improvement from 68.8 ± 14.7 to 72.4 ± 12.5, suggesting sustained emotional and cognitive functioning (*p* > 0.05). Scores in the social domain showed little fluctuation, whereas the environmental domain improved significantly (+4.2, *p* < 0.05), reflecting better perceptions of safety, resources, and living conditions. Overall, these findings suggest that despite chemotherapy, participants largely maintained their quality of life, with the most notable improvement observed in environmental well-being.

### 3.3. Body Composition Analysis Along with Physical Activity and Quality of Life

The mean body weight of the participants in the initial assessment was 69.5 ± 12.4 kg, ranging from 45.6 to 95.4 kg. In the second assessment, a statistically significant increase was observed, with a mean value of 70.5 ± 12.4 kg (range 47.6–98.3 kg; *p* = 0.001, Student’s *t*-test). This change was also reflected in the body mass index (BMI), which rose from an average of 26.6 ± 5.1 to 26.9 ± 5.0 (*p* = 0.001). Similarly, the fat-free mass index (FFMI) increased significantly from 17.6 ± 1.9 to 17.8 ± 1.8 (*p* = 0.011). Of particular note was the rise in muscle mass: total muscle mass increased from 43.8 ± 4.6 kg to 44.4 ± 4.7 kg (*p* = 0.001). These changes were especially pronounced in specific body segments, including the right leg (7.2 ± 0.8 vs. 7.3 ± 0.8 kg; *p* = 0.002), left leg (7.1 ± 0.8 vs. 7.2 ± 0.8 kg; *p* = 0.023), left arm (2.2 ± 0.3 vs. 2.3 ± 0.3 kg; *p* = 0.001), and trunk (25.0 ± 2.5 vs. 25.3 ± 2.7 kg; *p* = 0.032). By contrast, no significant differences were found in fat content. Whole-body fat percentage remained virtually unchanged, at 32.5 ± 7.6% in the first measurement and 32.7 ± 7.4% in the second (*p* = 0.874). Similarly, fat content in the trunk remained stable (28.6 ± 8.5% vs. 28.8 ± 8.0%; *p* = 0.918). Only in the right arm was a slight but statistically significant increase observed (30.6 ± 11.0% vs. 31.5 ± 10.6%; *p* = 0.041). In summary, the findings demonstrated a tendency toward increased muscle mass and higher BMI, with relative stability in total fat content (Table 2).

The relationships between body composition and physical activity, measured using the IPAQ-L questionnaire, revealed further noteworthy observations. In the first assessment, the correlations between variables were generally weak and statistically non-significant. However, in the second assessment, stronger associations emerged. Average sitting time correlated positively with body weight (rho = 0.282; *p* = 0.029) and BMI (rho = 0.256; *p* = 0.048). The strongest relationship was observed between sitting time and muscle mass (rho = 0.368; *p* = 0.004), suggesting that women with greater lean body mass tended to spend more time in sedentary positions. Additionally, a negative correlation was found between waist-to-hip ratio and vigorous physical activity (rho = −0.257; *p* = 0.048), indicating that women engaging in higher-intensity activity displayed more favorable body proportions. Other variables, including fat content and overall physical activity levels, did not demonstrate statistically significant associations with body composition parameters (Table 3).

A further dimension of the study was the analysis of associations between body composition and quality of life as assessed by the WHOQOL-BREF questionnaire. In the initial assessment, higher fat percentage was negatively correlated with both the physical domain (rho = −0.266; *p* = 0.040) and the social relationships domain (rho = −0.256; *p* = 0.049). These results indicate that greater fat mass was linked to poorer perceptions of physical functioning and social well-being. BMI also showed a negative correlation with the psychological domain (rho = −0.299; *p* = 0.020), suggesting that overweight and obesity were associated with lower psychological well-being. In the second assessment, the significance of fat distribution became more evident: WHR correlated negatively with the psychological domain (rho = −0.308; *p* = 0.017), emphasizing the potential impact of abdominal adiposity on mental health outcomes. Importantly, muscle mass did not show statistically significant relationships with any of the quality-of-life domains (*p* > 0.05), which may suggest that psychosocial and metabolic factors exert stronger influence than lean mass per se. Overall, the findings indicate that both the quantity and distribution of fat tissue negatively affect self-perceived quality of life among women after chemotherapy (Table 4).

## 4. Discussion

The present study investigated changes in body composition and their associations with physical activity and quality of life (QoL) in 60 women following chemotherapy for breast cancer. Our results revealed a statistically significant increase in body weight, BMI, and muscle mass, while fat mass remained relatively stable. Importantly, excess adiposity was negatively associated with multiple QoL domains, particularly physical, psychological, and social functioning. These findings are consistent with, but also extend, previous reports in the field.

In our cohort, mean body weight increased from 69.5 ± 12.4 kg to 70.5 ± 12.4 kg (*p* = 0.001), accompanied by a rise in BMI from 26.6 ± 5.1 to 26.9 ± 5.0 (*p* = 0.001). Fat-free mass index (FFMI) also increased significantly (17.6 ± 1.9 to 17.8 ± 1.8; *p* = 0.011), while total muscle mass rose from 43.8 ± 4.6 kg to 44.4 ± 4.7 kg (*p* = 0.001). These results echo the findings of Porciúncula Frenzel et al., who reported in a cohort of 70 women that chemotherapy induced a significant increase in body weight (*p* = 0.02), BMI (*p* = 0.03), and fat-free mass (*p* < 0.001) [10]. However, while Frenzel and colleagues observed that overall QoL remained unchanged during treatment, our study found negative correlations between fat mass and QoL domains, suggesting that body composition changes may exert subtle but meaningful effects on survivorship well-being. The observed increase in muscle mass during chemotherapy may be partly explained by changes in body water distribution detected by bioelectrical impedance analysis (BIA), rather than by true hypertrophy. Fluid retention, often seen during cytotoxic therapy, can artificially elevate lean mass estimates. This interpretation aligns with reports suggesting that early-phase BIA measurements should be interpreted cautiously due to potential hydration shifts.

Our analysis further showed that fat percentage was negatively correlated with physical QoL (rho = −0.266; *p* = 0.040) and social relationships (rho = −0.256; *p* = 0.049), while BMI was inversely correlated with psychological QoL (rho = −0.299; *p* = 0.020). Notably, waist-to-hip ratio (WHR) was significantly associated with lower psychological scores (rho = −0.308; *p* = 0.017) in the second assessment, highlighting the particular relevance of fat distribution. Similar findings were reported by Pavlović Mavić et al., who studied 68 premenopausal women undergoing chemotherapy. Their results indicated that body composition was strongly correlated with physical and sexual functioning at baseline, while later in treatment it was associated with fatigue and gastrointestinal side effects [11]. Collectively, these findings emphasize that the adverse psychosocial burden of excess adiposity is not limited to the early post-treatment phase but persists and may even intensify over time. Physical activity emerged as another key determinant in our study. Although most associations with body composition were weak, vigorous activity was inversely related to WHR (rho = −0.257; *p* = 0.048), and sedentary time correlated positively with BMI (rho = 0.256; *p* = 0.048) and muscle mass (rho = 0.368; *p* = 0.004). Although several correlations reached statistical significance, their strength (rho ≈ 0.25–0.30) indicates weak associations. Therefore, the clinical significance of these findings should be interpreted with caution. These findings suggest that central adiposity may be mitigated by more intense exercise, whereas sedentary behavior may reinforce unfavorable body composition changes. This may align with results by Hojan et al., who reported that aerobic training reduced fat body mass, and resistance training further increased fat-free body mass in premenopausal women receiving endocrine therapy [12]. In their cohort (n = 41), aerobic exercise alone decreased android fat percentage, while the addition of resistance training not only further reduced adiposity but also increased muscle mass and improved QoL scores. Thus, while our study observed only correlational associations, interventional studies such as Hojan’s provide causal evidence supporting exercise as a modifier of chemotherapy- and endocrine-related body composition changes. The stronger association between psychological QoL and obesity indicators may reflect the multidimensional burden of body image disturbance, reduced self-esteem, and cancer-related fatigue. Adiposity, particularly central fat distribution, can amplify emotional distress through both metabolic (inflammatory) and psychosocial pathways, explaining why psychological well-being was more strongly affected than physical or social domains.

Evidence from more recent interventional trials further substantiates these observations. Isanejad et al. compared high-intensity interval training (HIIT) and moderate-intensity continuous training (MICT) in 30 breast cancer patients during endocrine therapy [13]. VO_2_ peak improved by 16.8% in the HIIT group, while both HIIT and MICT significantly improved HDL cholesterol and multiple QoL subdomains. Importantly, emotional well-being improved in both groups (HIIT mean difference +4.41, MICT + 4.25, both *p* < 0.05), while functional well-being improved only in HIIT (+3.35, *p* < 0.05). These improvements occurred without significant changes in body weight or BMI, underscoring that exercise may enhance QoL even in the absence of marked anthropometric changes. By contrast, our study showed that fat mass and BMI were directly related to poorer QoL outcomes, highlighting the dual importance of both exercise-induced functional gains and adiposity reduction.

Meta-analytic evidence supports this dual perspective. Joaquim et al. synthesized 12 systematic reviews including up to 5761 patients, finding that exercise interventions significantly improved QoL in 83.3% of included studies, with additional benefits for cardiorespiratory fitness and reductions in waist circumference [14]. Similarly, Yang et al. reported in a meta-analysis of 548 overweight/obese survivors that exercise interventions significantly reduced BMI by −1.37 kg/m^2^ and body fat by −3.8% compared with routine care [4]. While improvements in mental health were not statistically significant in their pooled analysis, our results demonstrate that both BMI and fat percentage were associated with lower psychological well-being, suggesting that longer interventions or more targeted exercise regimens may be required to produce robust mental health benefits.

Our findings also resonate with the work of Courneya et al., who examined 1458 newly diagnosed patients and found that women in the lowest quartile of lean mass percentage were at more than twice the risk of poor physical QoL (OR = 2.31, 95% CI 1.37–3.89) compared to those in the highest quartile [15]. Although muscle mass in our cohort increased modestly (by 0.6 kg overall, *p* = 0.001), it did not directly correlate with QoL. This discrepancy may be due to differences in timing (newly diagnosed vs. post-chemotherapy) or to the relatively narrow range of lean mass variation in our sample.

Beyond exercise, dietary interventions also warrant attention. Kämmerer et al. conducted a non-randomized trial in 152 women, showing that both low-carbohydrate and ketogenic diets improved QoL, physical performance, and muscle-to-fat ratio, with ketogenic diets producing the most favorable improvements in metabolic health [16]. Such results, in conjunction with our findings on the adverse effects of adiposity, point to the potential utility of combining dietary and exercise interventions in survivorship programs.

Finally, the clinical implications of adiposity extend beyond QoL. Ligorio et al. highlighted that overweight and obesity negatively affect prognosis in HER2-positive breast cancer, potentially by fostering systemic inflammation and resistance to anti-HER2 therapies [17]. While our study did not assess survival outcomes, the strong associations we observed between adiposity and diminished QoL reinforce the need for targeted lifestyle interventions not only to enhance survivorship well-being but also to potentially improve long-term oncological outcomes.

In summary, our results demonstrate that while chemotherapy is associated with increases in muscle mass and BMI, excess fat and unfavorable fat distribution remain key determinants of reduced QoL. These findings, taken together with the broader body of evidence, strongly advocate for the integration of structured exercise and dietary programs into post-treatment care. Such interventions may not only counteract adverse body composition changes but also enhance physical, psychological, and social well-being, ultimately improving both survivorship experience and prognosis. Although some changes in quality of life did not reach statistical significance, the direction and magnitude of observed effects may hold clinical relevance. Even small improvements in environmental and psychological well-being can translate into better treatment adherence and functional recovery. Therefore, our results should be interpreted in both statistical and clinical contexts. Future studies should prioritize structured, combined interventions integrating exercise and dietary counseling, possibly supplemented with psychological support. Extending observation beyond six weeks and including varying exercise intensities could better capture the trajectory of recovery and its impact on quality of life.

This study has several limitations. The relatively small sample size and lack of a control group restrict the generalizability of the findings. Additionally, reliance on self-reported physical activity data may introduce recall bias. The observation period of approximately six weeks reflects only the early phase of chemotherapy, which may limit the ability to capture long-term trends. These factors should be addressed in future, larger-scale randomized or controlled studies.

## 5. Conclusions

This pilot study indicates that even early during chemotherapy, body composition and QoL are interrelated. While exercise and dietary interventions are likely beneficial, our findings emphasize the need for structured, individualized programs that combine moderate-intensity physical activity, dietary counseling, and psychological support. Future studies should test these interventions in randomized settings to determine their true efficacy.

## Figures and Tables

**Table 1:** WHOQOL-BREF results (N = 60).

	First Assessment				Second Assessment				*p*-Value *
Domains	M	SD	Min.–Max.	% n ≥M	M	SD	Min.–Max.	% n ≥M	
**Physical**	54.7	12.7	25.0–75.0	45%	54.2	10.5	38.0–69.0	51.7%	0.798
**Psychological**	68.8	14.7	21.0–94	71.7%	72.4	12.5	19.0–100.0	56.7%	0.113
**Social relationships**	51.4	8.0	6.0–56.0	51.7%	51.5	7.7	25.0–56.0	63.3%	0.788
**Environment**	81.5	13.9	44.0–100.0	45%	85.7	12.7	38.0–100.0	58.3%	0.028

M—mean, Min.–Max.—Minimal to maximal value, SD—Standard deviation, %n ≥ M—percentage of people who scored higher than or equal to the mean value, *—Wilcoxon test.

**Table 2:** Body composition analysis (N = 60).

	First Assessment					Second Assessment					Assessment Difference (II–I)	*p*-Value
	M ± SD	Min–Max	Me	Q1	Q3	M ± SD	Min–Max	Me	Q1	Q3		
**Body mass [kg]**	69.5 ± 12.4	45.6–95.4	69.5	60.5	76.9	70.5 ± 12.4	47.6–98.3	70.8	62.5	78.1	1.0	0.001 **
**Waist circumference [cm]**	92.9 ± 10.3	71.0–117.0	92.0	84.0	100.5	93.5 ± 9.8	71.0–140.0	93.0	84.0	102.0	0.1	0.539 **
**Hip circumference [cm]**	109.0 ± 11.6	73.0–133.0	108.0	102.0	117.0	108.6 ± 12.4	75.0–134.0	108.0	100.5	116.5	−0.4	0.564 **
**Arm circumference [cm]**	32.7 ± 7.5	25.0–81.0	31.0	29.0	35.0	33.5 ± 8.1	24.0–73.0	31.5	30.0	35.0	0.8	0.209 *
**WHR**	0.86 ± 0.11	0.72–1.54	0.84	0.80	0.89	0.87 ± 0.16	0.72–1.87	0.85	0.81	0.89	0.01	0.564 *
**BMI**	26.6 ± 5.1	17.7–41.8	25.8	22.8	29.6	26.9 ± 5.0	17.8–41.1	26.4	23.3	29.9	0.3	0.001 **
**FFMI**	17.6 ± 1.9	14.5–24.4	17.4	16.1	18.5	17.8 ± 1.8	13.9–23.5	17.6	16.5	19.0	0.2	0.011 *
**ABSI**	0.08 ± 0.005	0.07–0.10	0.08	0.07	0.08	0.08 ± 0.008	0.06–0.13	0.08	0.07	0.08	0.0	0.085
**Fat content [%]**	32.5 ± 7.6	15.3–44.3	33.1	27.5	38.8	32.7 ± 7.4	13.9–46.6	33.4	28.4	37.7	0.2	0.874 *
**Muscles [kg]**	43.8 ± 4.6	34.6–52.9	42.6	40.1	47.0	44.4 ± 4.7	35.4–54.3	44.8	41.3	48.3	0.6	0.001 **
**Right leg**												
**Fat content [%]**	38.4 ± 6.3	25.2–51.3	38.9	34.2	43.4	38.2 ± 6.7	20.7–20.7	39.4	52.2	33.6	−0.2	0.536 **
**Muscles [kg]**	7.2 ± 0.8	5.8–8.9	7.2	6.6	7.8	7.3 ± 0.8	5.6–9.1	7.5	6.8	7.8	0.1	0.002 **
**Left leg**												
**Fat content [%]**	38.5 ± 6.1	26.0–49.9	39.0	34.8	43.1	38.7 ± 6.2	25.9–50.9	39.7	34.3	42.6	0.2	0.307 **
**Muscles [kg]**	7.1 ± 0.8	5.6–9.0	7.0	6.5	7.5	7.2 ± 0.8	5.6–8.9	7.2	6.6	7.7	0.1	0.023 **
**Right arm**												
**Fat content [%]**	30.6 ± 11.0	7.4–52.4	31.5	23.3	38.7	31.5 ± 10.6	6.9–51.8	31.9	25.2	39.1	0.9	0.041 **
**Muscles [kg]**	2.3 ± 0.3	1.7–3.1	2.2	2.0	2.5	2.3 ± 0.3	1.7–3.1	2.3	2.1	2.4	0.0	0.282 **
**Left arm**												
**Fat content [%]**	32.5 ± 10.5	11.0–54.3	32.6	26.1	40.5	32.8 ± 10.4	6.9–53.6	32.5	26.8	40.6	0.3	0.543 **
**Muscles [kg]**	2.2 ± 0.3	1.6–2.9	2.2	2.0	2.5	2.3 ± 0.3	1.6–3.0	2.3	2.1	2.5	0.1	0.001 **
**Trunk**												
**Fat content [%]**	28.6 ± 8.5	6.9–41.8	29.9	22.5	35.3	28.8 ± 8.0	7.0–45.5	30.0	24.7	34.1	0.2	0.918 *
**Muscles [kg]**	25.0 ± 2.5	19.9–29.7	24.6	22.8	27.1	25.3 ± 2.7	19.3–30.9	25.5	23.5	27.2	0.3	0.032 *

ABSI—A Body Shape Index, BMI—Body Mass Index, FFMI—Fat-Free Mass Index, M—Mean, Me—Median, Min.–Max.—Minimal to maximal value, Q1—First Quartile, Q3—Third Quartile, SD—Standard deviation, WHR—Waist-to-Hip Ratio, * Wilcoxon test, ** Student’s *t*-test.

**Table 3:** The relationship between body composition analysis and physical activity (N = 60).

Body Composition Variables	IPAQ-L Domains	First Assessment		Second Assessment	
		Spearman’s Rho	*p*-Value	Spearman’s Rho	*p*-Value
**Body mass [kg]**	Total walking	−0.034	0.794	−0.126	0.337
	Total moderate activity	0.171	0.189	0.145	0.268
	Total vigorous activity	−0.052	0.689	−0.149	0.256
	Total physical activity	0.129	0.328	0.019	0.882
	Total time sitting	−0.047	0.722	0.203	0.120
	Average total time sitting	−0.013	0.922	0.282	**0.029**
**BMI**	Total walking	−0.048	0.712	−0.073	0.578
	Total moderate activity	0.104	0.428	0.073	0.577
	Total vigorous activity	−0.090	0.493	−0.153	0.244
	Total physical activity	0.064	0.629	0.019	0.881
	Total time sitting	−0.026	0.846	0.169	0.195
	Average total time sitting	0.011	0.933	0.256	**0.048**
**WHR**	Total walking	−0.142	0.281	−0.095	0.469
	Total moderate activity	−0.120	0.360	0.145	0.268
	Total vigorous activity	−0.184	0.159	−0.257	**0.048**
	Total physical activity	−0.128	0.328	0.003	0.979
	Total time sitting	0.023	0.861	0.141	0.282
	Average total time sitting	0.073	0.578	0.131	0.318
**Fat content [%]**	Total walking	−0.001	0.992	−0.062	0.636
	Total moderate activity	0.238	0.067	0.021	0.876
	Total vigorous activity	−0.150	0.251	−0.178	0.175
	Total physical activity	0.173	0.185	−0.038	0.772
	Total time sitting	−0.047	0.719	0.035	0.793
	Average total time sitting	0.000	0.995	0.109	0.405
**Muscles [kg]**	Total walking	−0.079	0.546	−0.169	0.195
	Total moderate activity	0.134	0.307	0.198	0.129
	Total vigorous activity	0.094	0.475	−0.064	0.626
	Total physical activity	0.064	0.625	0.055	0.677
	Total time sitting	0.006	0.965	0.288	**0.026**
	Average total time sitting	0.001	0.992	0.368	**0.004**

BMI—Body Mass Index, FFMI—Fat-Free Mass Index, IPAQ-L—International Physical Activity Questionnaire—Long Form, WHR—Waist-to-Hip Ratio.

**Table 4:** The relationship between body composition analysis and quality of life (N = 60).

Body Composition Variables	WHOQOL-BREF Domains	First Assessment		Second Assessment	
		Spearman’s Rho	*p*-Value	Spearman’s Rho	*p*-Value
**Body mass [kg]**	Physical	−0.169	0.196	−0.189	0.149
	Psychological	−0.249	0.055	−0.088	0.503
	Social relationships	−0.169	0.195	−0.131	0.319
	Environment	−0.197	0.131	−0.091	0.486
**BMI**	Physical	−0.235	0.070	−0.163	0.213
	Psychological	−0.299	**0.020**	−0.078	0.552
	Social relationships	−0.213	0.102	−0.107	0.415
	Environment	−0.220	0.091	0.031	0.817
**WHR**	Physical	−0.029	0.827	−0.233	0.073
	Psychological	−0.035	0.793	−0.308	**0.017**
	Social relationships	−0.169	0.198	−0.140	0.285
	Environment	−0.132	0.316	−0.162	0.215
**Fat content [%]**	Physical	−0.266	**0.040**	−0.151	0.248
	Psychological	−0.249	0.054	−0.101	0.442
	Social relationships	−0.256	**0.049**	−0.032	0.809
	Environment	−0.210	0.107	−0.012	0.929
**Muscles [kg]**	Physical	0.042	0.748	−0.076	0.564
	Psychological	−0.086	0.513	−0.065	0.621
	Social relationships	0.074	0.574	−0.114	0.386
	Environment	−0.031	0.815	−0.095	0.473

BMI—Body Mass Index, FFMI—Fat-Free Mass Index, WHOQOL-BREF—World Health Organization Quality of Life—BREF, WHR—Waist-to-Hip Ratio.

## Data Availability

The data presented in this study are available on request from the corresponding author due to privacy and ethical restrictions.

## Abbreviations

The following abbreviations are used in this manuscript:

ACSM	American College of Sports Medicine
ABSI	A Body Shape Index
BMI	Body Mass Index
FFMI	Fat-Free Mass Index
HER2	Human Epidermal Growth Factor Receptor 2
HIIT	High-Intensity Interval Training
IPAQ-L	International Physical Activity Questionnaire–Long Form
MICT	Moderate-Intensity Continuous Training
QoL	Quality of Life
SD	Standard Deviation
WHOQOL-BREF	World Health Organization Quality of Life–BREF
